# Supplementary material for: Novel Characterization Techniques for Multifunctional Plasmonic–Magnetic Nanoparticles in Biomedical Applications
Source: Nanomaterials (Basel). 2023 Nov 11;13(22):2929. doi: 10.3390/nano13222929 (PMC10675523; doi:10.3390/nano13222929)
Supplement: Supplementary file 1 [file nanomaterials-13-02929-s001.zip › nanomaterials-2701564-supplementary.pdf]

Supplementary Material

Novel Characterization Techniques for Multifunctional Plasmonic–Magnetic Nanoparticles in Biomedical Applications

Rodrigo Calvo [1], Isabel Rodriguez Mariblanca [1], Valerio Pini [1,*], Monica Dias [1], Virginia Cebrian [1], Andreas Thon [1], Asis Saad [1], Antonio Salvador-Matar [1], Óscar Ahumada [1], Miguel Manso Silván [2], Aaron E. Saunders [3], Wentao Wang [4] and Adonis Stassinopoulos [4]

[1] Mecwins S.A., Tres Cantos, 28760 Madrid, Spain.
[2] Departamento de Física Aplicada, Universidad Autónoma de Madrid, Campus de Cantoblanco, 28049 Madrid, Spain.
[3] nanoComposix™, San Diego, CA 92111, USA.
[4] QuidelOrtho™,San Diego, CA 92121, USA.

* Correspondence: vpini@mecwins.com

Quantitative Elemental Mapping using X-ray Energy Dispersive Spectroscopy (EDS)

Using X-ray energy dispersive spectroscopy (EDS), we can create a map that shows the concentration of specific elements in a sample. The process begins with a beam of electrons directed at the sample. As these electrons interact with the surface, they cause its atoms to become excited and emit specific X-rays. Each element emits X-rays at specific, unique energies that appear as peaks in an EDS spectrum.

By systematically scanning the electron beam across the particles, an EDS spectrum is collected at each point. To determine the concentration of a particular element, the intensity of its characteristic peak in each of these spectra is monitored.

Quantitative mapping with X-ray energy dispersive spectroscopy (EDS) involves scanning a sample with an electron beam in a two-dimensional fashion. This produces EDS spectra at each position. Within these spectra there are characteristic peaks such as Au Mα1 (around 2.13 keV), O Kα1 (around 0.5 keV) and Fe Lα1 (close to 0.95 keV) as shown in Figure S1. a. The intensity of these peaks is normalized and then a correction is made taking into account the presence of other elements and a reference spectrum, but some times the tail of higher peaks over-shadows the presence of other element peak, as for example Au. The result is a detailed map showing, for each pixel, the exact concentration of the element of interest in the sample, as shown in Figure S1.b.

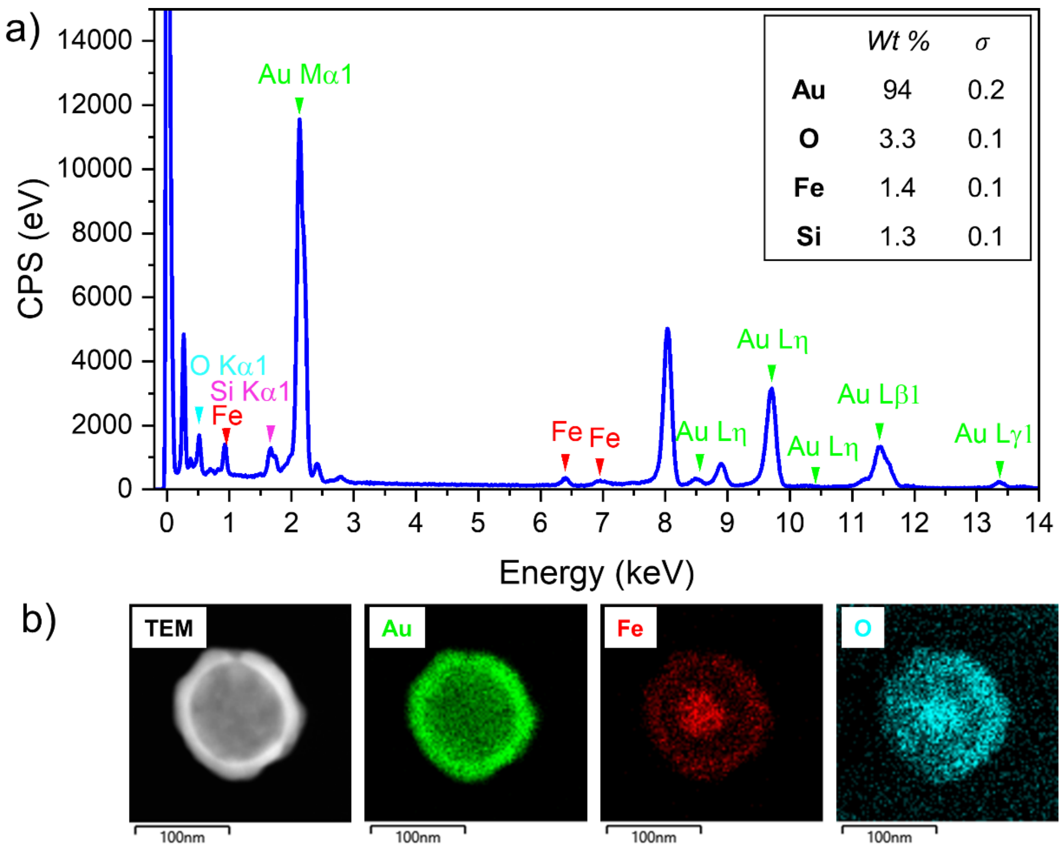

Figure S1: (a) EDS spectrum showing characteristic peaks for various elements, with prominent peaks for Au Mα1, O Kα1, Fe Lα1, and other noted transitions. The inset table shows the weight percentages and standard deviations for the detected elements. (b) Transmission electron microscopy (TEM) image of the sample (left) accompanied by quantitative EDS elemental maps showing the spatial distribution of gold (Au, green), iron (Fe, red), and oxygen (O, cyan). Each elemental map is scaled to 100 nm.
